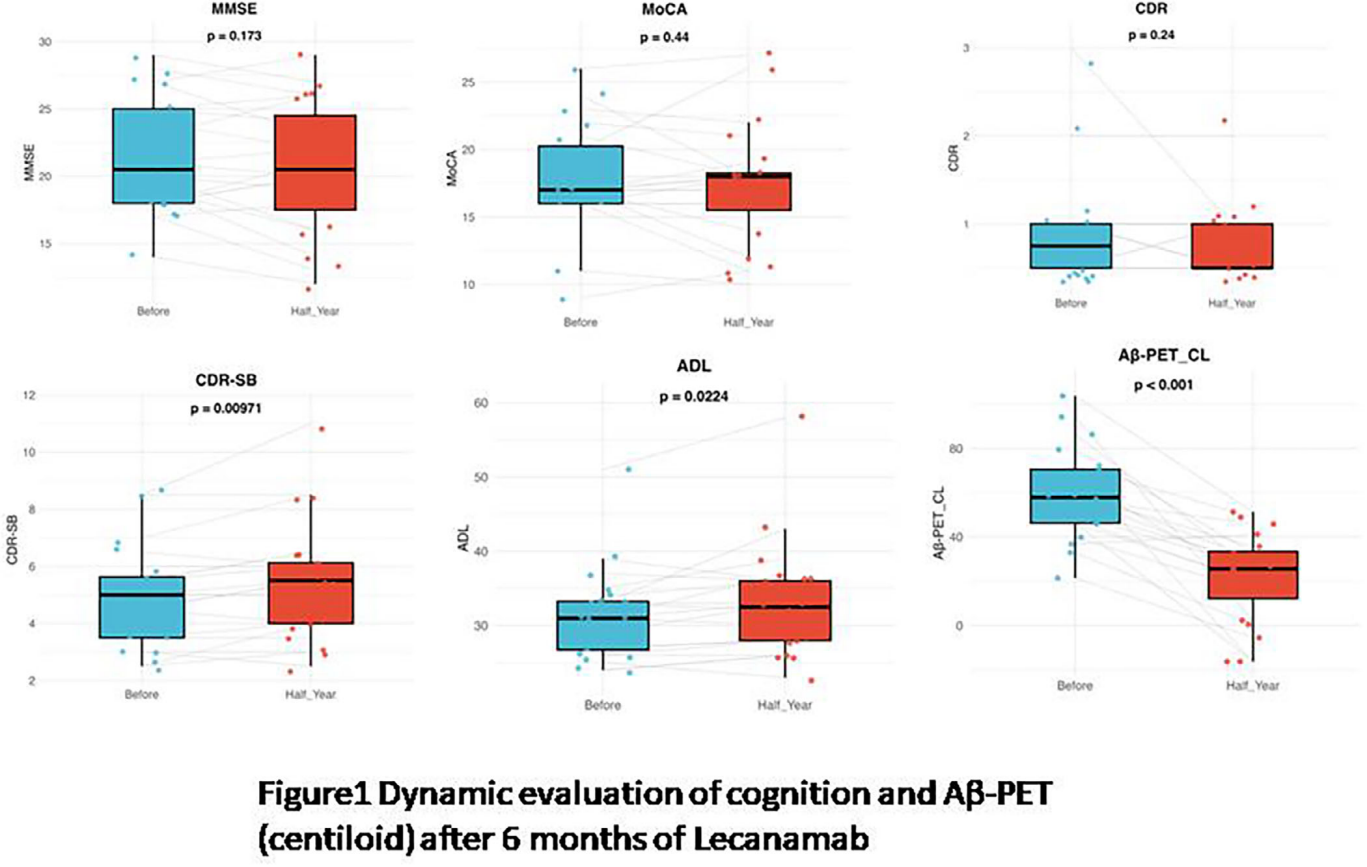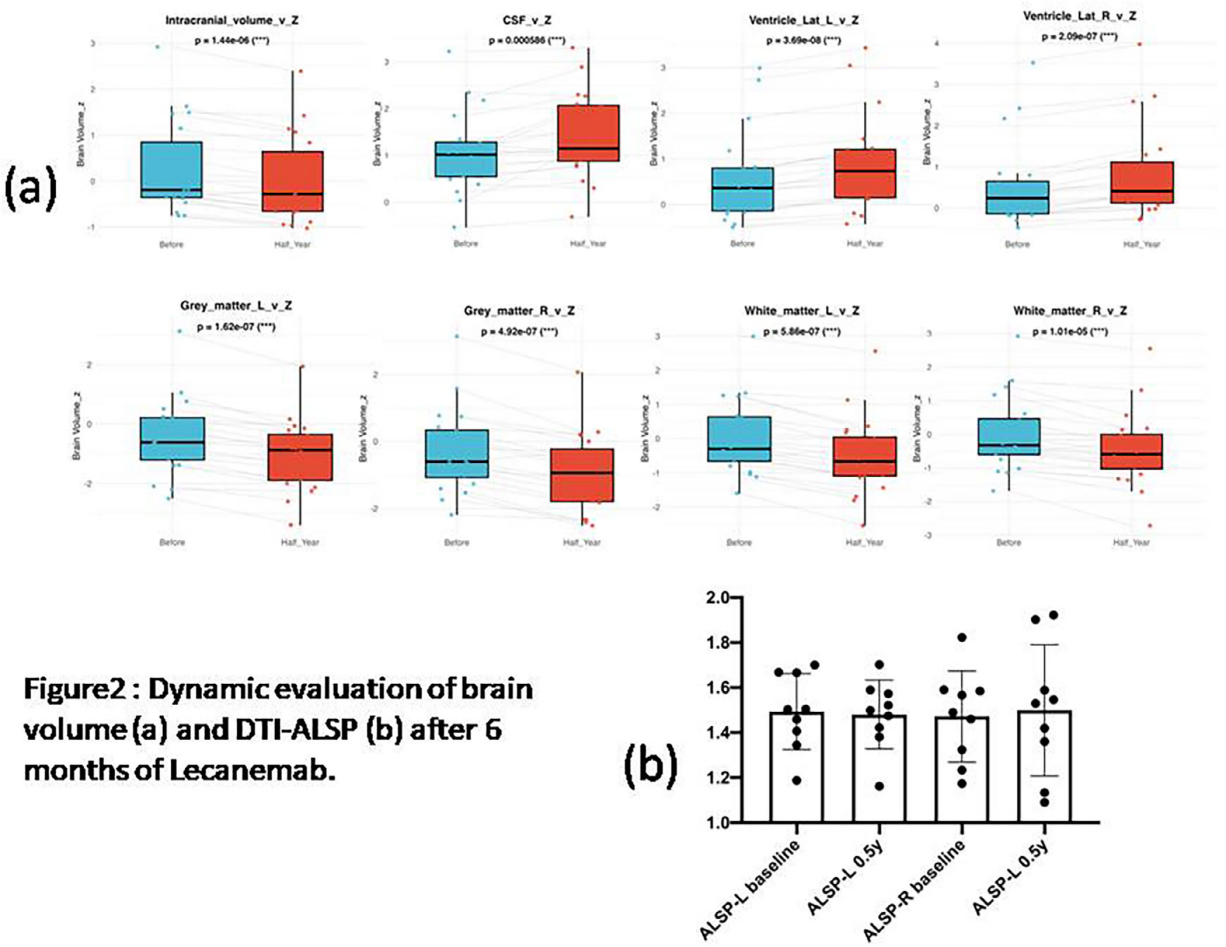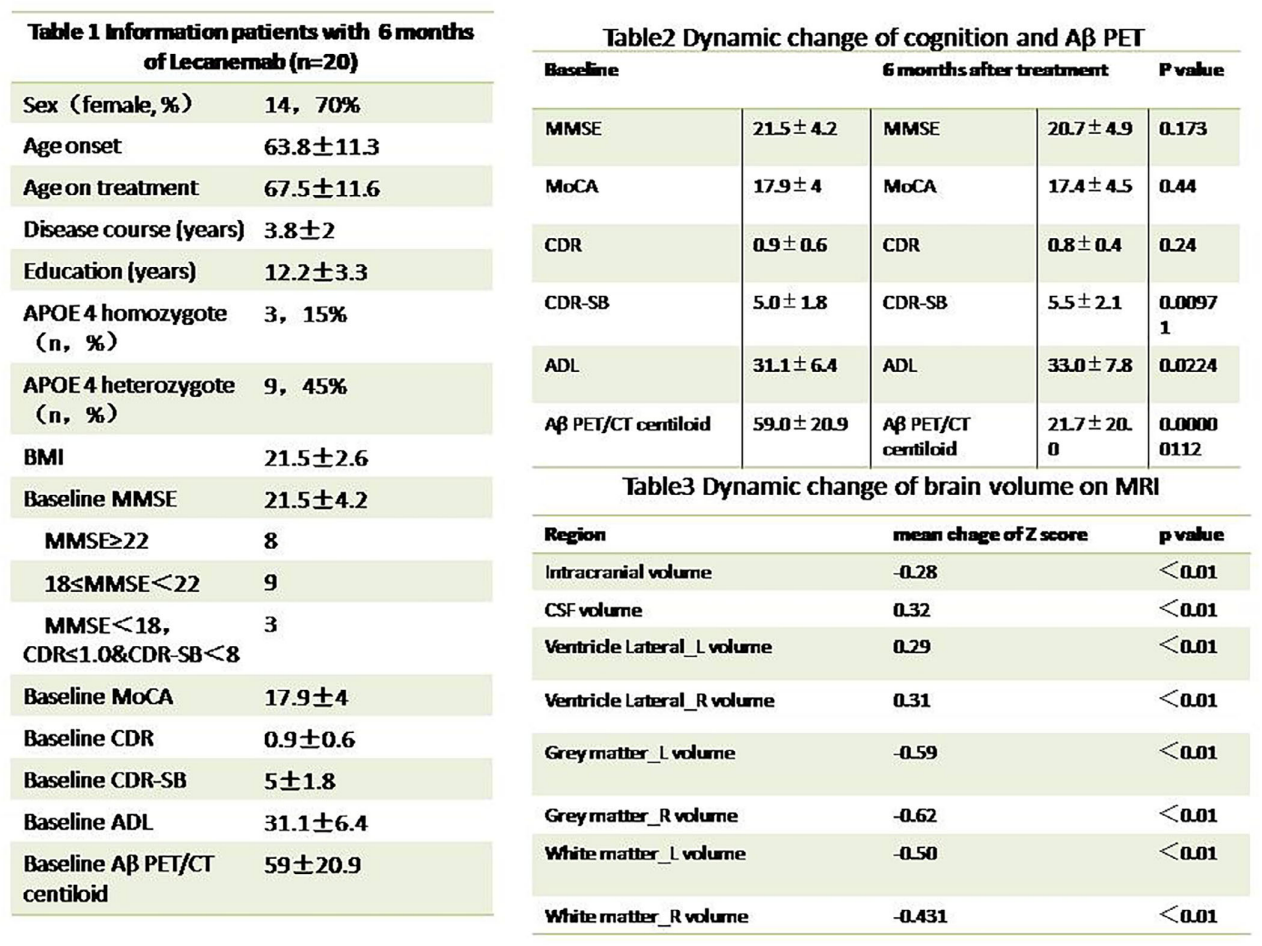# Real‐World Clinical Practice of Lecanemab at PUMCH: Focus on Dynamic Imaging and Biomarker Evolution

**DOI:** 10.1002/alz70861_108414

**Published:** 2025-12-23

**Authors:** Chenhui Mao, Wenjun Wang, Xinying Huang, Meiqi Wu, Yifei Wang, Tianyi Wang, Yuyue Qiu, Shanshan Chu, Wei Jin, Yuhan Jiang, Jialu Bao, Yunfan You, Yuanheng Li, Liling Dong, Dongjing Li, Xinhua Wan, Feng Feng, Li Huo, Ling Qiu, Jing Gao

**Affiliations:** ^1^ Peking Union Medical College Hospital, Beijing, Beijing China; ^2^ Peking Union Medical College Hospital, Beijing China

## Abstract

**Background:**

Lecanemab was widely used in China since June 2024 . Its real‐world efficacy and safety profile have attracted significant attention. The aim of the study is to evaluate the clinical outcomes, adverse effects, and dynamic follow‐up results of lecanemab in a Chinese population.

**Method:**

The study enrolled AD patients treated with lecanemab at Peking Union Medical College Hospital (PUMCH), all confirmed by cerebrospinal fluid (CSF) and/or PET imaging (Aβ: AV45 or PiB; Tau: MK6240). Baseline assessments included demographic data collection, APOE genotyping, and comprehensive cognitive evaluations. MRI protocols comprised 3D T1, T2 FLAIR, SWI, T2, DWI, and DTI sequences. Adverse events were documented. At the 6‐month follow‐up, cognitive function, MRI, and Aβ‐PET were reassessed (Centiloid quantification). Brain volumetrics were analyzed using United Imaging software (Z‐scores derived). Plasma biomarkers (Aβ42, Aβ40, *p* ‐tau217, *p* ‐tau181) were measured quarterly via LUMIPULSE‐G to track longitudinal trends.

**Result:**

The cohort comprised 42 patients (M:F=12:30; age range 48‐89 years [≤65:16; >65:26]). APOE ε4: wild‐type (15), heterozygous (17), and homozygous (8). Baseline MMSE 8‐29 (CDR 0.5‐1). The safety outcomes include: ARIA‐E (1 case), ARIA‐H (1 case), ARIA‐E+H (1 case) – all asymptomatic without treatment interruption; Infusion‐related fever (6 cases, 14.3%); Other transient events (9 cases: insomnia, transient hypertension, headache); 1 central retinal vein occlusion and 1 elevated creatinine with unclear drug relationship. Efficacy in 20 Patients Completing 6‐Month Follow‐up was evaluated (Table 1). Cognitive Function remained stable (Table 2 + Figure 1). Aβ‐PET Centiloid decreased significantly from 59.0±20.9 to 21.7±20.0 (*p* <0.001, Figure 1). Significant reductions in total brain, gray/white matter volumes; ventricular/CSF volume expansion were found (Table 3 + Figure 2a). No significant DTI‐ALSP changes found in 9 cases (Figure 2b). Among 19 tau‐PET stratified patients (low burden:4; moderate‐high:15), no differential treatment response was observed (possibly due to small sample size). Plasma biomarkers evaluation: Aβ42/Aβ40 ratio (baseline:0.0667 → 6mo:0.0708); *p* ‐tau217 (0.7801→ 0.762 pg/mL); *p* ‐tau181 (2.3426→ 2.1478 pg/mL). While statistical significance was not reached, improving trends were found.

**Conclusion:**

Six‐month lecanemab treatment demonstrated Clinical stabilization with robust amyloid clearance, MRI brain volume reduction, preserved glymphatic function, promising biomarker trends requiring longer observation and favorable safety profile in Chinese patients.